# Asymmetric expression level of clock genes in left vs. right nasal mucosa in humans with and without allergies and in rats: Circadian characteristics and possible contribution to nasal cycle

**DOI:** 10.1371/journal.pone.0194018

**Published:** 2018-03-13

**Authors:** Ha Kyun Kim, Hyun Jung Kim, Jae Hyung Kim, Tae Hoon Kim, Sang Hag Lee

**Affiliations:** Department of Otorhinolaryngology-Head & Neck Surgery, College of Medicine, Korea University, Seoul, South Korea; University of Lübeck, GERMANY

## Abstract

Numerous peripheral tissues possess self-sustaining daily biologic rhythms that are regulated at the molecular level by clock genes such as *PER1*, *PER2*, *CLOCK*, *and BMAL1*. Physiological function of nasal mucosa exhibits rhythmic variability to a day-night environmental cycle. Nevertheless, little is known of the expression and distribution pattern of clock genes in nasal mucosa. The present study investigates the expression level and distribution pattern of *PER1*, *PER2*, *CLOCK*, *and BMAL1* genes in nasal mucosa of healthy controls, allergic rhinitis patients, and normal rats. In human and rat nasal mucosa, the levels of these genes are asymmetrically expressed in nasal mucosa derived from right and left cavities in normal controls, allergic patients, and rat. In human nasal mucosa, the expression levels of these genes were higher in the decongested side than the congested mucosa. In rat nasal mucosa, these clock genes are expressed in a rhythmic circadian manner under the regular light/dark cycles. The expression levels of MUC5AC, a key mucin genes produced in superficial epithelium, are higher in decongested side than that congested side in human nasal mucosa. In rat nasal mucosa, MUC5AC levels showed a circadian rhythm which was associated with different expression levels in nasal mucosa derived from the right and left nasal cavities. Taken together with these results, the present study shows that the clock genes such as *PER1*, *PER2*, *CLOCK*, *and BMAL1* are present in human and rat nasal mucosa, and suggest that these clock genes may control the pathophysiological function of nasal mucosa as circadian oscillators and affect the maintenance of the nasal cycle.

## Introduction

Spontaneous periodic changes in the congestion state of the nasal mucosa associated with reciprocal fluctuations in nasal resistance to airflow are referred to as the nasal cycle. Cyclical shrinkage and dilation of the venous sinusoids distributed in the nasal mucosa contributes to the spontaneous alteration in nasal airflow [1–5]. This physiological phenomenon is present in 80% of normal human subjects and is also found in several animal species [5–9]. Although the function of the nasal cycle has not been clearly established, the cyclical changes in the congestion state of the nasal mucosa associated with nasal cycle may result in plasma extravasation and nasal fluid formation [3]. During the nasal cycles, one nasal cavity becomes more open, and its mucosal glands increase their secretion, while nasal mucosa in the opposite cavity becomes engorged, which diminishes mucosal gland secretion [10, 11]. Eccles proposed that the nasal cycle may have a role in respiratory defense by alternating air conditioning between the two nasal passage and by generating the nasal fluid [3]. Furthermore, nasal cycle are accompanied by change in dilation of the venous sinuses distributed in the nasal turbinate and nasal septum. The dilation state is controlled by the autonomic nervous system that regulates vasomotor activity of the nasal mucous membranes [9, 12]. It is believed that the action of the sympathetic nerve controlling reciprocal changes of nasal airflow is regulated by the vasomotor control areas of the medulla [13, 14]. The hypothalamus participates in regulation of the nasal cycle via the sympathetic nervous system distributed in the nasal mucosa [15].

Data reported in numerous studies have shown that many mucosal functions in nasal cavities are subject to a circadian rhythm. In normal subjects, a prominent circadian fluctuations in concentration of IgG, IgA, and albumin was revealed in nasal secretions [16, 17]. Circadian changes of the mucociliary transport times have also been shown [18]. Concentrations of albumin, elastase, and IL-8 in nasal lavage samples from healthy volunteers were significantly different between morning and afternoon [19]. The amount of collected nasal secretion was significantly higher at 6 a.m. than at 3 p.m. after a metacholine nasal challenge. Furthermore, numerous studies showed the diurnal manifestation and 24-h variation in the severity of allergic rhinitis [20, 21]. Symptoms of allergic rhinitis are relatively intense nocturnally, during sleep, and in the early morning [22]. A statistically higher concentration of inflammatory mediators was detected in the nasal secretions of allergic rhinitis patients after a challenge in the morning than in the afternoon. Such a circadian variation of nasal reactivity suggests that the ability to induce inflammatory activity in nasal mucosa depends on a circadian rhythm [23]. However, the regulator of these circadian rhythms at the molecular level has yet to be determined.

The molecular basis of the biological circadian rhythm is believed to be determined by the clock genes, which are expressed within the suprachiasmatic nucleus as well as in the peripheral tissues [24–29]. The molecular analysis of the biological clock genes has revealed that a basic mechanism of the clock machinery composes the autoregulatory positive and negative feed-back loops of a set of clock genes [30, 31]. These feedback loops are composed of the basic helix-loop-helix transcription factors CLOCK and BMAL1. These molecules dimerize, and activate transcription of the Period genes *Per1* and *Per2* [31–35]. Recent studies have revealed circadian variations in expression of clock genes in peripheral tissues, indicating that the analysis of clock gene expression profiles in peripheral tissues may provide useful clues for the elucidation of their individual circadian functions [24, 36, 37].

In this respect, we hypothesized that clock genes may be rhythmically expressed in nasal mucosa. Therefore, the present study had the following aims:

1. to investigate the expression levels and distribution pattern of *PER1*, *PER2*, *CLOCK*, and *BMAL1* genes in normal and allergic human nasal mucosa and to establish whether their levels correlate temporally with alternating congestion and decongestion of nasal mucosa;
2. to elucidate whether these genes exhibit asymmetrical expression levels in nasal mucosa of opposite nasal cavities in rat and whether their levels are subject to a circadian rhythm;
3. to determine if the expression levels of these genes are associated with the production of MUC5AC and aquaporin 5.

## Materials and methods

### Subjects & sample preparation

This study was approved by the Ethics Committee of Korea University and The Committee of Care and Use of Laboratory Animals of Korea University. Normal control subjects and patients with moderate/severe persistent allergic rhinitis were selected for this study. The clinical characteristics of normal controls and allergic patients are presented in Table 1. Patients who were admitted to hospital for augmentation rhinoplasty were considered as normal control subjects. Normal controls did not have any significant clinical symptoms as revealed by a physical examination, nasal examination, and medical history. Moderate/severe persistent allergic rhinitis was defined according to the Allergic Rhinitis and Its Impact on Asthma criteria [38]. All subjects were nonsmokers without any pulmonary diseases and were not taking any medication during the preceding 3 months.

**Table 1:** Clinical characteristics of normal control and allergic patients.

Characteristics	Normal Control	Allergic patients
**Female/Male**	8/16	5/8
**Age, yr**	34.9±16	31.2±16.2
**Rhinitis duration**	NA	12.58±7.28
**Serum total IgE, IU/ml**	52.64±21.36	469±224.86

Mean ± SD

Acoustic rhinometry was performed in all subjects enrolled in this study in the morning between 7:00 AM and 8:00 AM. Immediately after measurement of nasal cavity volume, nasal mucosa was respectively sampled from both inferior turbinate under local anesthesia. This study was approved by the institutional ethics committee, and all participants gave written informed consent.

All tissue samples were divided into three parts. The first and second parts were stored at –70 °C for subsequent RNA and protein isolation. The third part of samples was used for immunohistochemistry.

### Animal experiment

The Committee of Care and Use of Laboratory Animals of our institution approved all animal care and experimental procedure. Experimental procedures for animals are reported in accordance with the Animal Research Reporting of In Vivo Experiments (ARRIVE) guidelines [39, 40].

Male 5–6 week old Sprague-Dawley rats (DBL, Chungcheong Buk-Do, Korea) were maintained at a temperature of 23 °C ± 2°C under a 12 light-dark cycle per day. Animals were sacrificed every 3 h using an anesthetic overdose (Ketamine, 15 mg per 100g and xylazine, 2 mg per 100g, intraperitoneal administration) at each time point 3h apart during two consecutive 24 h spans in total darkness beginning at the time of usual lights-on at 09:00 AM (Zeitgeber Time 0, where ZT0 is lights-on. ZT12 is lights-off). Thereafter, nasal mucosa was obtained from both nasal cavities and kept in a deep freezer for isolation of mRNA and protein. For immunohistochemistry, nasal mucosa was fixed in 4% paraformaldehyde and then rinsed with 30% sucrose.

### RT-PCR and real-time RT-PCR analysis

Nasal mucosa samples were analyzed using RT-PCR and real time PCR for *PER1*, *PER2*, *CLOCK*, *BMAL1*, *MUC5AC*, *and aquaporin 5* (for primers see Table 2). Total RNA was extracted from each sample using TRIzol reagent (Invitrogen, Burlington, ON, CA) and used for cDNA synthesis in a reaction mixture containing 2.5 U of MML-V (GIBCO BRL, Grand Island, N.Y., USA). Thereafter, resulting cDNA was amplified by RT-PCR or real time PCR.

**Table 2:** Sequences of PCR primers.

Primer	Sequence	
**hPER1**	S: 5′- AGGTGAGAGTAGCGGAGAG -3′	AS: 5′- AGGGAGAGGGCAGGTTAG -3′
**hPER2**	S: 5′- CAGCCTTTCGACTATTCTCCC -3	AS: 5′- TGACTTTGTGCCTCCCAATG -3′
**hCLOCK**	S: 5′- GTCCCAGTTTCAGTTTTCAGC -3′	AS: 5′- TCTATCATGCGTGTCCGTTG -3
**hBMAL1**	S: 5′- TCAGTGATTTCATGTCCCCG -3′	AS: 5′- CATTGTGCTCCCCAAATTCG -3′
**hMUC5AC**	S: 5′-CAGCCACGTCCCCTTCAATA -3′	AS: 5′- ACCGCATTTGGGCATCC -3′
**hAquaporin**	S: 5′- GAATCTACTTCACTGGCTGC -3′	AS: 5′- GGTAGAAGTAAAGGATGGCAG -3′
**hGAPDH**	S:5′- CCACATCGCTCAGACACCAT -3′	AS: 5′- AGTTAACAGCCCTGGTGA -3′
**hEEF1A1**	S:5′-GTGTTCCTTTGGTCAACACCG-3′	AS: 5′-ACAACCCTATTCTCCACCCAG-3′
**rPER1**	S: 5′- CACATCTGAATACACTCTCCG -3	AS: 5′- GCTCCGAAATATAGACAATCCG -3′
**rPER2**	S: 5′- GCGTTCCCTTATGTGGTTTG -3′	AS: 5′- ACTAAGCTCCATCAGTCCAG -3′
**rCLOCK**	S: 5′- CAAGGAAGCACTGGAAAGG -3′	AS: 5′- CGTTGAGGAAGGGTCTGAG -3′
**rBMAL1**	S: 5′- CACCAACCCATACACAGAAG -3′	AS: 5′- GACAGATTCGGAGACAAAGAG -3′
**rMUC5AC**	S: 5′- ACTCTATCCACATCTACCGC-3′	AS: 5′- TCTCCCCTTTCAGTCTTGG -3′
**rAquaporin**	S: 5′- CCTTATCCATTGGCTTGTCTG -3′	AS: 5′- AATAGGCCCTACCCAGAAG -3′
**rGAPDH S:**	S: 5′- GCCTTCTCTTGTGACAAAGTG -3′	AS: 5′- TGACTGTGCCATTGAACTTG -3′
**rEEFA1**	S: 5′- TGTTGTGAAAGCCACCGCTA -3′	AS: 5′- TTGCCGGAATCTACGTGTCC -3′

h; human, r; rat

Real-time PCR was carried out in a Bio-Rad iCycler using SYBR green. Amplification was conducted after a 30-s denaturation at 95°C followed by 45 cycles of incubation at 95°C for 15 s and at 60°C for 20 s. All reactions were performed in triplicate, and expression of each target gene was normalized to *GAPDH* or EEF1A1expression. The relative amount of target gene in each sample was determined as 2 ^ΔΔCt^.

### Immunohistochemical and western blot analysis

To investigate the localization of clock genes protein in human and rat nasal mucosa, tissue sections were incubated overnight at 4°C with a mouse monoclonal anti-PER1 antibody (1: 200 dilution, sc-81574), a mouse monoclonal anti-PER2 antibody (1: 200 dilution, sc-377290), a mouse monoclonal anti-CLOCK antibody (1:100 dilution, sc-271603), or a mouse monoclonal anti-BMAL1 antibody (1:100 dilution, sc-373955), (all antibodies were from Santa Cruz Biotechnology, Inc., Santa Cruz, CA, USA). As a negative control, the primary antibodies were replaced with nonimmune normal serum (Santa Cruz Biotechnology). Diaminobenzidine (DAB; Sigma-Aldrich, St. Louis, MO, USA) was used for color development.

For Western blot analysis, the extracted protein from frozen tissue samples was resolved on 4–12% Tris glycin SDS-PAGE gels and transferred to Immobilon (Millipore, Bedford, Mass., USA). The blots were blocked with PBS-T containing 1% skim milk and then incubated with anti-PER1, anti-PER2, anti-BMAL1 (all diluted to 1:500), anti-CLOCK antibody (diluted to 1: 200), or a mouse monoclonal anti-aquaporin 5 (1;100 dilution, sc-514022) (Santa Cruz Biotechnology, Inc., Santa Cruz, Calif., USA) in PBS-T overnight at room temperature. As an internal control, β-actin expression level was measured in parallel blots using a β-actin antibody (Santa Cruz Biotechnology, Inc., Santa Cruz, Calif., USA). The intensity of detected bands was quantified using Scion Image Beta 4.0.2

### Data analysis

Statistical analyses were carried out using SPSS for Windows (version 16.0.0; SPSS, Chicago, Ill., USA). The expression levels of clock genes in normal and allergic patients analyzed by two-way analysis (ANOVA). Associations between the expression levels of genes and rhinometric data were analyzed using the Mann-Whitney U test. The level of significance was set at p < .05. If significant group differences were detected by the ANOVA, then a post hoc analysis was applied. Cosinor analysis was then performed using fold increase over ZT0 to test for the presence of a circadian rhythm. Changes in clock gene expression were referenced to the levels of expression at the 9 AM. time point (ZT0). Data are presented as means ± SD (n = 6 mice per time point).

## Results

**Fig 1:**
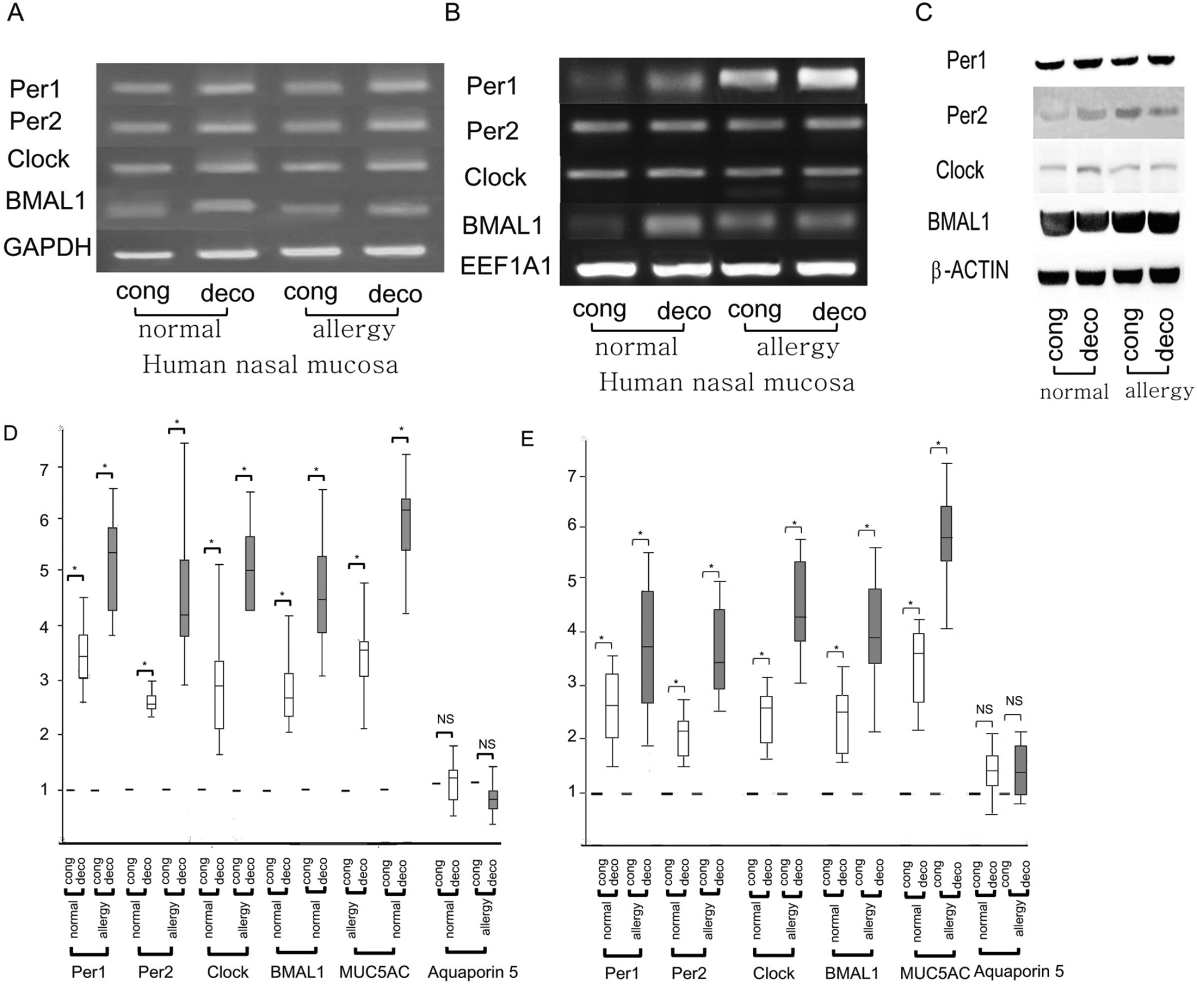
**Representative panels showing the expression of *PER1*, *PER2*, *CLOCK*, and *BMAL1* in congested (cong) and decongested (deco) nasal mucosa of normal controls (n = 24) and allergic rhinitis patients (n = 13), which were evaluated by RT-PCR (A, B) and western blot (C)**. The expression levels of *PER1*, *PER2*, *CLOCK*, *and BMAL1* genes using GAPDH (A, D) or EEF1A1 (B, E) as housekeeping gene in congested (cong) and decongested (deco) mucosa of normal controls (white bar) and allergic patients (grey bar) are compared by using real time PCR (D, E). * p <0.05; significantly different as indicated. NS; not significant.

**Fig 2:**
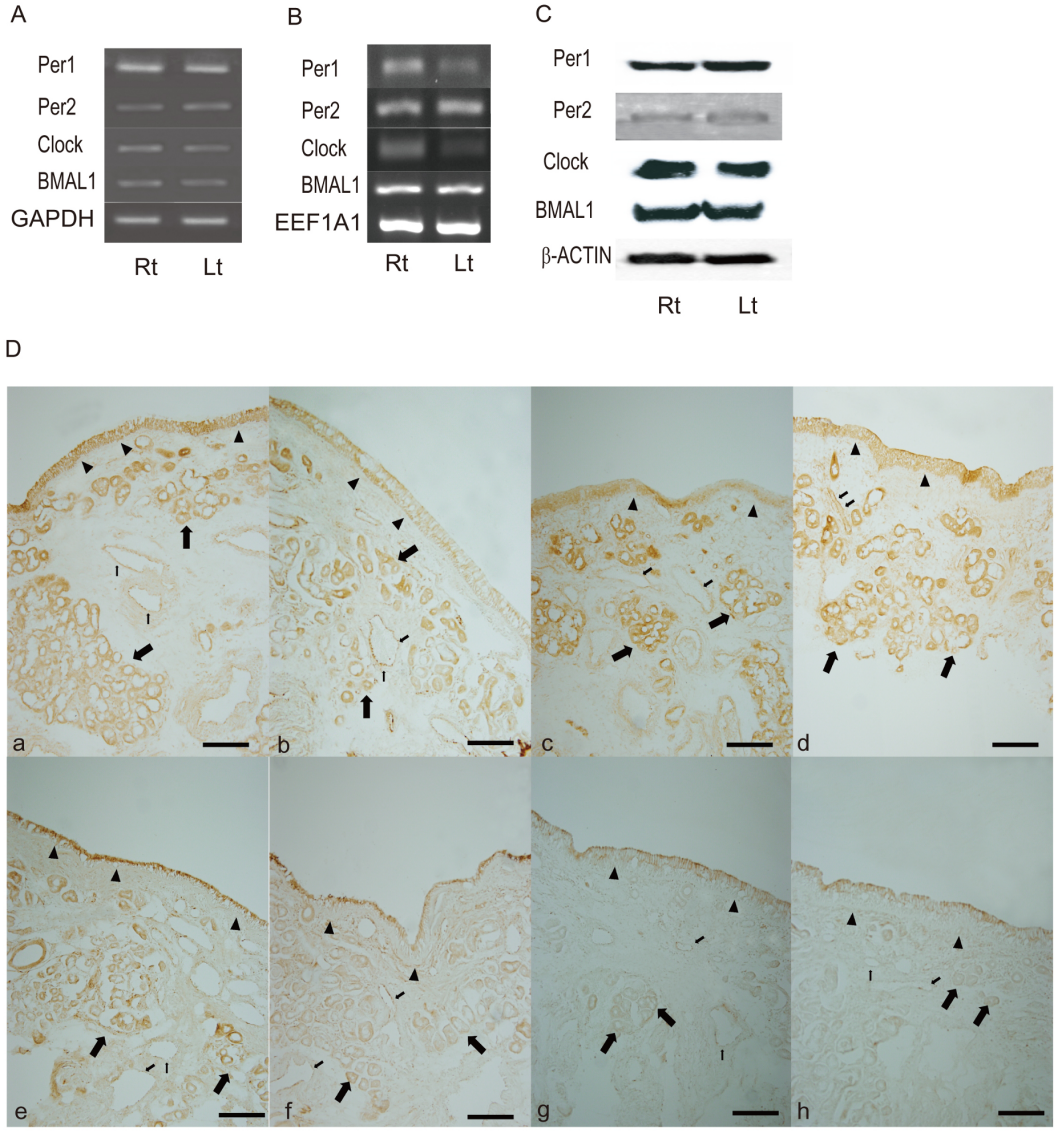
**Representative panels showing the expression of *PER1*, *PER2*, *CLOCK*, and *BMAL1* in nasal mucosa derived from the right (Rt) and the left (Lt) nasal cavity of rat which were evaluated by RT-PCR (A and B; A indicates the usage of GAPDH as housekeeping genes, B shows the EEF1A1 as housekeeping gene) and western blot (C)**. (D) Immunohistochemistry shows the distribution of *PER1* (a, b), *PER2* (c, d), *CLOCK* (e, f), and *BMAL1* (g, h) in normal (a, c, e, and g) and allergic nasal mucosa (b, d, f, and h). Arrow head indicates superficial epithelium. Large arrow; submucosal glands. Small arrow; vascular endothelium. Scale bar indicates 50 uM.

RT-PCR performed with primers specific for *PER1*, *PER2*, *CLOCK*, *and BMAL1* mRNA showed that these genes are expressed in normal nasal mucosa of rat as well as in normal and allergic human nasal mucosa (Figs 1A, 1B and 2). Real-time PCR was conducted to evaluate the expression levels of *PER1*, *PER2*, *CLOCK*, *and BMAL1* genes in total RNA isolated from nasal mucosa of both inferior turbinate of the human nasal cavity. Interestingly, these results revealed that these genes had asymmetric expression levels in nasal mucosa from both turbinates in normal control and allergic patients and showed that the expression levels of these genes were higher in decongested mucosa than congested mucosa, irrespective of GAPDH or EEF1A1 usage as housekeeping gene (Fig 1D and 1E). Asymmetric expression levels of these genes were found in all subjects enrolled in the present study.

*PER1*, *PER2*, *CLOCK*, *and BMAL1* protein expression in the human nasal mucosa was confirmed by western blot (Fig 1C). Expression of these proteins in all samples was also confirmed by immunohistochemistry. Immunoreactivity of these genes was detected in normal and allergic human nasal mucosa, where *PER1*, *PER2*, *CLOCK*, *and BMAL1* localized to the epithelial cells, submucosal glands, and vascular endothelium (Fig 2D).

We then evaluated the possibility that asymmetric expression levels of these genes may be related to the nasal cycles, particularly to congested or decongested phase evaluated by acoustic rhinometry. The expression levels of *PER1*(F = 25.7, P <0.05), *PER2* (F = 6.6, P <0.05), *CLOCK* (F = 8.7, p<0.05), *and BMAL1* (F = 10.8, p<0.05) genes were higher in decongested mucosa than in congested mucosa in normal controls and allergic patients (Fig 1D and 1E). However, the levels of these clock genes expressed in decongested mucosa were not different between normal and allergic patients (Fig 1D and 1E).

**Fig 3:**
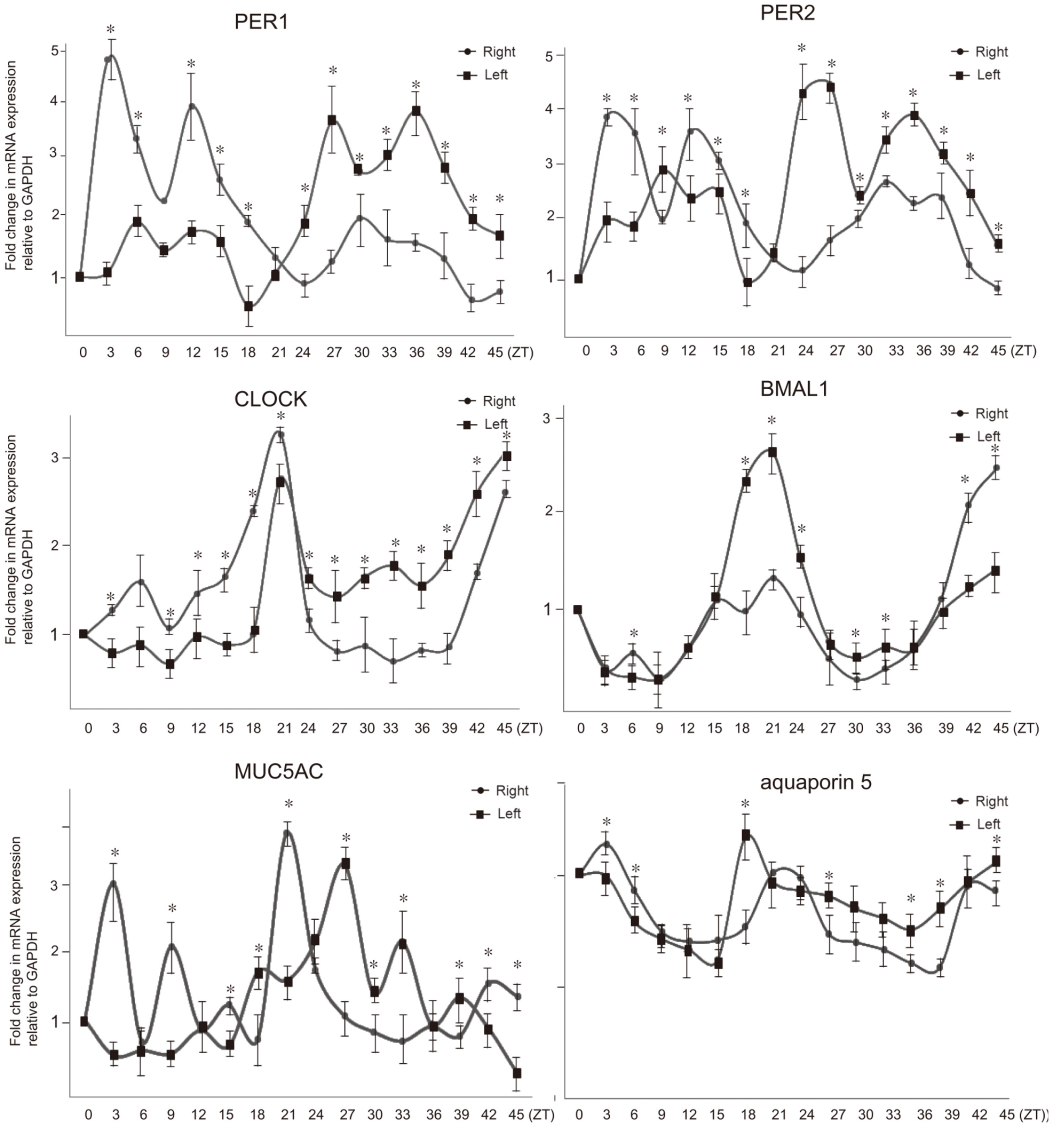
Daily profiles of *PER1* (A), *PER2* (B), *CLOCK* (C), *BMAL1* (D), *MUC5AC* (E), and aquporin5 (F) mRNA levels in nasal mucosa derived from the right and the left nasal cavity of rat under light/dark conditions. Zeitgeber Time (ZT) is used to assess endogenous time in constant darkness. ZT0 and ZT24 is assigned to the original time of lights on and ZT12 and ZT 36 to light off. Values of *PER1*, *PER2*, *CLOCK*, *BMAL1*, *MUC5AC*, *and aquaporin* mRNA were normalized to GAPDH mRNA. The signal levels at ZT0 were defined as 1. Each value represents the means ± SD (n = 6 mice per time-point). The difference of expression levels were assessed by Mann-Whitney U test. * means P < .05. All time interval calculations are based at the indicated Zeitgeber time (ZT), and 9:00 a.m. was considered ZT0.

**Fig 4:**
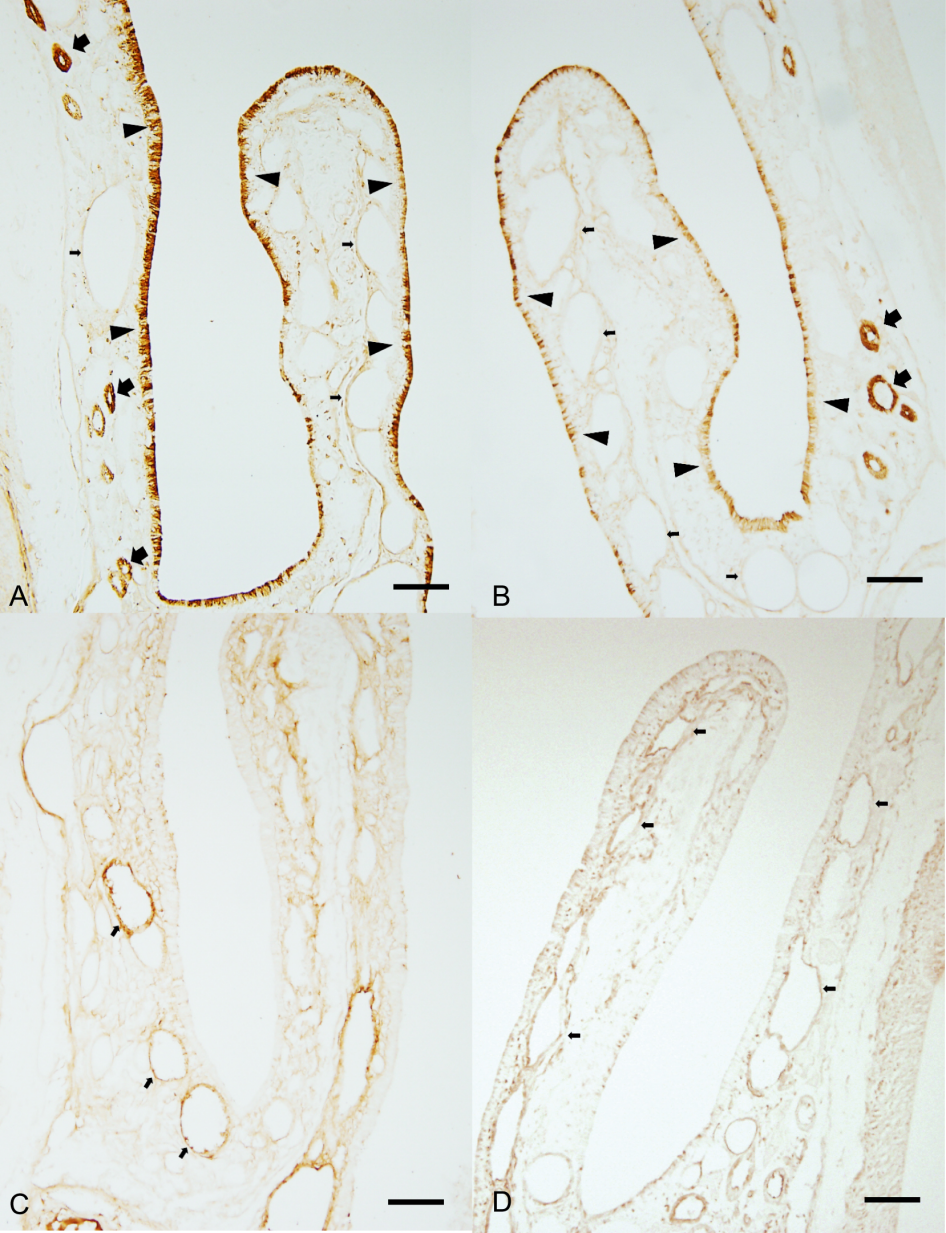
Representative pictures show the distribution of *PER1* (A) of right nasoturbinate, *PER2* (B) of left nasoturbinate, *CLOCK* (C) of right nasoturbinate, and *BMAL1* (D) of left nasoturbinate of rat at CT6. Arrow head indicates superficial epithelium, large arrow; submucosal gland, small arrow: vascular endothelium. Scale bar indicates 50 uM.

Rat nasal mucosae obtained from both nasal cavity of rat showed also asymmetric expression in mRNA and protein levels of *PER1*, *PER2*, *CLOCK*, *and BMAL1* (Fig 2A, 2B and 2C). Rhythmic expression patterns for *PER1*, *PER2*, *CLOCK*, *and BMAL1* genes were observed for two consecutive days in the nasal mucosa of rat (Fig 3). Multiple peaks in *PER1* mRNA levels in mucosa from the right nasal cavity appears at ZT3 and ZT12 (95% CI, cosinor, p<0.001) (Fig 3A). Then PER1 mRNA levels decreased at ZT24 before increasing again at ZT30 (95% CI, cosinor, p<0.001). In contrast, PER1 mRNA level in mucosa from the left nasal cavity was low until ZT18. Thereafter, its levels rose, showing a double peak at ZT27 and ZT36 (95% CI, cosinor, p < 0.001) (Fig 4A). *PER2* mRNA levels in mucosa from the right nasal cavity peaked at ZT3 and ZT12 (95% CI, cosinor, p< 0.001) (Fig 3B), demonstrating a double peak, and then declined between ZT21 and ZT27. Double peaks also were found on the 2^nd^ day at ZT33 and ZT39 (95% CI, cosinor, p<0.045) In mucosa from the left nasal cavity, PER2 mRNA level dynamics were opposite to those detected in right nasal cavity. *CLOCK and BMAL1* mRNA levels in mucosa from the right and left nasal cavities showed peaks at ZT21 and ZT45 (95% CI, cosinor, p< 0.036), and then, their mucosal levels were reversed between both nasal mucosa, showing different expression levels between both nasal mucosa. The expression levels of *PER1*, *PER2*, *and CLOCK* genes were significantly different in mucosa from the right and left nasal cavities suggesting their asymmetrical expression levels in the two nasal cavities. However, although the mucosal expression levels of *BMAL1* genes in the right and left nasal cavities were different during peak times, no statistical differences in BMAL1 expression were noted during the decreased circadian phase (Fig 3A, 3B, 3C and 3D). These antiphase expression patterns between *PER1/PER2* and *CLOCK/BMAL1* suggest that there is a functional peripheral circadian core oscillator in the nasal mucosa of rat. Furthermore, immunohistochemical experiments also showed that the expression of protein products of these genes was found in the respiratory mucosa of rat nasal cavity. In contrast to their expression patterns in the human nasal mucosa, Rat *PER1* and *PER2* proteins were predominantly distributed in the superficial epithelial layer and submucosal glands, whereas rat *CLOCK* and *BMAL1* proteins were mainly localized to the vascular endothelium (Fig 4).

To evaluate whether the expression levels of *MUC5AC* and aquaporin 5 genes may be associated with circadian expression levels of clock genes in human and rat nasal mucosa, real time PCR and western blots were performed. The results of these experiments showed that *MUC5AC* expression levels were higher in the decongested side in human nasal mucosa (Fig 1A, 1B, 1D and 1E) and were subject to circadian rhythm in the rat nasal mucosa (Fig 3E). In contrast, although aquaporin 5 gene levels showed circadian rhythms in rat nasal mucosa, but its levels were not different between the right and left nasal cavities during several circadian time points (Fig 3F). Furthermore, aquaporin 5 levels were not different between congested and decongested sides in the human nasal mucosa. (Fig 1D and 1E).

## Discussion

In this study, we present molecular and immunologic evidence of circadian clock-genes in the human and rat nasal mucosa. Notably, *PER1*, *PER2*, *CLOCK*, and *BMAL1* were asymmetrically expressed in nasal mucosa derived from the right and left nasal cavities of normal control and allergic patients. The expression levels of these clock genes were higher in decongested mucosa than in congested mucosa in normal control and allergic rhinitis. Asymmetric expression of these four clock genes was also found in nasal mucosa derived from the left and right nasal cavities of rat, each clock gene showing a circadian rhythm. In rat nasal mucosa, *PER1* and *PER2* expression levels showed a robust rhythm and were accompanied by antiphase oscillations in *CLOCK* and *BMAL1* levels. These findings provide evidence that a functional circadian clock is present in human and rat nasal mucosa, acting as distinct, circadian oscillators to set the circadian rhythm. Furthermore, the present results suggest the possibility that asymmetric expression of clock genes may be associated with the nasal cycle.

Numerous studies have reported that clock genes are expressed in peripheral human tissues or cells. Clock genes in those cell function as peripheral oscillators of the circadian rhythm, [41–46]. In this respect, the existence of a circadian clock within nasal mucosa was anticipated because numerous studies reported circadian variations in the pathophysiological functions of nasal mucosa. Although circadian variation in human biopsy samples could not be determined, expression levels of all clock genes tested in the present study were subject to robust circadian rhythms in rat nasal mucosa under light-dark cycle conditions. Based on these results, we conclude that clock genes are expressed in human and rat nasal mucosa. Moreover, a circadian rhythm in nasal mucosa is likely to function independently of the central clock distributed in the brain. Our results are consistent with previous results that showed significant circadian variation in the levels of *PER1* and *PER2* mRNA in mouse nasal mucosa, where *PER2* was localized to the epithelial cells, vascular endothelial cells, and nerve termini [47]. In the present study, the cellular localization of the four clock proteins was different in human and rat nasal mucosa. In human nasal mucosa, positive immunoreactivity of four clock genes was mainly detected in the superficial epithelium, submucosal glands, and vascular endothelial cells. However, in rat nasal mucosa, strong immunoreactivity of *PER1* and *PER*2 was mainly observed in the superficial epithelium and in a small number of submucosal glands, whereas the vascular endothelium was only lightly stained. In contrast, *CLOCK* and *BMAL1* were mainly localized to the vascular endothelium. Based on their characteristic localization pattern, we hypothesized that these clock genes may play their divergent roles in the physiological function of human and rat nasal mucosa. Because clock proteins in human nasal mucosa were identified in the superficial epithelium, submucosal gland, and vascular endothelium, it is conceivable that their expression might be involved in circadian rhythm of mucosal function such as nasal secretion and blood supply of microvasculature. In rat nasal mucosa, *PER1* and *PER2* might be involved in secretory function, whereas *CLOCK* and *BMAL1* genes might regulate blood supply. Further studies are warranted to determine whether there is clock gene-mediated interplay between blood flow and secretion.

It has been generally accepted that cyclical changes in nasal airway resistance are caused by autonomic tone that regulates blood supply to the mucosal vessels and is under the control of a hypothalamic center [15]. Nevertheless, the control process underlying the nasal cycle is not fully understood. Interestingly, we found that there are significant differences in the expression levels of clock genes in nasal mucosa in both human nasal cavities. The expression levels of these clock genes in decongested mucosa were higher than in congested mucosa. These results show that clock genes may possibly be involved in the nasal cycle of congestion and decongestion of the nasal mucosa. Similar findings were also noted in nasal mucosa of rat. It has been demonstrated previously that vessels in nasal mucosa are divided into resistance vessels and capacitance vessels [48]. The resistance vessels are composed of small arteries, veins and arterio-venous anastomosis. Especially, arterio-venous anastomosis, controlled by the autonomic nervous system, regulates nasal mucosa blood flow by alternating smooth muscle tone. In contrast, the major capacitance vessels in nasal mucosa are composed of vascular sinusoid, and its swelling is mainly affected by nasal mucosa blood flow and filling pressure. The volume of blood flow in nasal mucosa affects nasal resistance, and the dilation of vascular sinusoid can result in nasal congestion, contributing to nasal obstruction [49–53]. The existence of a circadian rhythm in the function of blood vessels has long been recognized. The clock genes are ubiquitously expressed and exhibit rhythmic expression in blood vessels. Cumulative evidence has demonstrated that these 24-h timers exerts a significant influence on the vasculature, whereby disruption of circadian clock components results in vascular stiffness and altered endothelial progenitor cell function [54]. Moreover, mutations of clock genes have been reported to have detrimental effects on the vascular function in mice [55]. Mice deficient in *BMAL1* and *CLOCK* genes showed a reduced vasodilatory response to acetylcholine and had impaired endothelial function [56]. Selective deletion of *BMAL1* from smooth muscle attenuated the circadian rhythm of blood pressure [57]. In this respect, we hypothesize that clock genes involved in the control of blood flow may contribute to the regulation of the nasal cycle. More studies are required to evaluate the molecular mechanisms of clock gene functions in cyclical congestion and decongestion of nasal mucosa.

Significant changes in the circadian amplitude of clock gene expression has been shown to cause cellular dysfunction and chronic diseases [58, 59]. The expression levels of clock proteins such as *BMAL1* and *PER2* are reduced in inflamed lung tissue and peripheral blood mononuclear cells in patients with chronic obstructive pulmonary disease [60]. Viral respiratory infection in the lung induced molecular clock dysfunction and increase mortality in *BMAL1* knockout mice, suggesting that altered clock function impairs the immune response [61]. Circadian alterations in expression levels of CLOCK, CRY1, and PER2 mRNA were found in schizophrenia [62]. The expression levels of *BMAL1* and *PER1* are normally in antiphase with each other, as demonstrated, for example, in the synovial membrane samples obtained from patients with osteoarthritis, where high *BMAL1* expression coexisted with low *PER1* expression. This expression pattern is commonly found in all clock works in the body [26]. However, this antiphase phenomenon was not found in rheumatoid arthritis patients. These results indicate that the clock is dysfunctional in rheumatoid arthritis, suggesting that inflammation may disturb circadian time-keeping [63]. In this respect, although the relationships between allergic rhinitis and clock genes functions remains unclear, we hypothesized that clock genes expression levels might be altered in allergic nasal mucosa in comparison with their levels in normal nasal mucosa. However, no significant differences in the expression levels of *PER1*, *PER2*, *CLOCK*, and *BMAL1* in human control and allergic nasal mucosa samples were noted in the present study. In a recent study, the expression pattern of clock genes showed a clear circadian rhythm with a peak at ZT13 in both control and asthmatic mice. No significant differences in the daily rhythmic pattern of *PER1* and *PER2* expression were observed in control and OVA-treated mice [64], which suggests that allergic responses in the lung may not interact with clock functions.

Recently, an association between the molecular circadian clock and the immune system and inflammation has been recognized. For example, passive cutaneous and systemic anaphylactic reactions were observed in wild-type, but not in *PER*2-mutant mice [65]. These reactions were also absent in mice with mechanical disruption of the central SCN clock [66]. Ear swelling, serum IgE levels, and the number of mast cells were significantly increased in Clock mutant mice, providing evidence that *CLOCK* mutation induces the T-helper type 2 immune response and aggravates contact hypersensitivity in skin [67]. Bone marrow-derived basophils in wild-type mice exhibited circadian variation in IgE-mediated IL-4 and histamine production, which was not observed in bone marrow-derived basophils of mice bearing a mutation in the *CLOCK* gene [68]. Therefore, functional analyses of resident cells including nasal epithelial cells or inflammatory cells infiltrated into nasal mucosa in clock genes mutant mice are further required.

Since robust rhythms of *PER1* and *PER2* were observed in superficial epithelial layer of rat nasal mucosa, we investigated whether the expression of genes encoding *MUC5AC* and aquaporin5, secretory substances produced in superficial epithelium, is subject to circadian rhythm in nasal mucosa. In human nasal mucosa, the expression of *MUC5AC* is higher in the decongested side than in congested side. In rat nasal mucosa, *MUC5AC* levels showed a circadian rhythm associated with different expression levels in the right and left nasal cavities. Expression levels of PER1 and PER2 was the highest at ZT3 and ZT12 on the right side and at ZT27 and ZT36 on the left side. MUC5AC levels was the highest at ZT3, ZT9, and ZT21 on the right side and at ZT27 and ZT33 on the left side. However, the expression levels of aquporin5 were not different between the two nasal cavities in human and rat nasal mucosa and exhibit a lower amplitude oscillation. These findings suggests that both *MUC5AC* and aquaporin5 production are modified by circadian change, but aquaporin5 oscillates with a lower amplitude. It is known that muscarinic M1, M2, M3 receptors are expressed in nasal mucosa [69, 70]. Because muscarinic receptors in the mouse lung show a circadian rhythm in their expression levels, and because in nasal mucosa, muscarinic type 3 receptors participate in mucin secretion [70, 71], it is speculated that the circadian regulation of *MUC5AC* secretion is controlled by the muscarinic receptors. Nevertheless, further experiments are needed to assess the circadian regulation of mucin secretion.

In conclusion, the present study revealed that the clock genes *PER1*, *PER2*, *CLOCK*, and *BMAL1* are present in human and rat nasal mucosa, displaying asymmetric expression levels between nasal cavities, and their expression levels were subject to circadian rhythms in rat nasal mucosa. These results suggest that the clock genes may play a role as circadian oscillators in nasal mucosa and affect the maintenance of the nasal cycle.
